# Perioperative safety and prognosis following parenchyma-preserving surgery for solid pseudopapillary neoplasm of the pancreas

**DOI:** 10.1186/s12957-023-03003-y

**Published:** 2023-03-31

**Authors:** Yong Gao, Feng Guo, Zipeng Lu, Chunhua Xi, Jishu Wei, Kuirong Jiang, Yi Miao, Junli Wu, Jianmin Chen

**Affiliations:** 1 Pancreas Center, The First Affiliated Hospital with Nanjing Medical University, Nanjing, Jiangsu 210029 People’s Republic of China; 2 Pancreas Center, The Affiliated BenQ Hospital of Nanjing Medical University, Nanjing, Jiangsu 210029 People’s Republic of China

**Keywords:** Pancreas, Solid pseudopapillary neoplasm, Surgical management, Parenchyma-preserving pancreatectomy, Prognosis

## Abstract

**Background/objectives:**

To evaluate perioperative safety and outcome of parenchyma-preserving pancreatectomy and risk factors of metastasis and recurrence for patients with solid pseudopapillary neoplasm (SPN).

**Methods:**

Demographic data, operative and pathological parameter, follow-up data of patients with SPN undergoing their first operation were collected in our single center from May 2016 to October 2021 and compared between regular pancreatectomy group and parenchyma-preserving surgery group. Risk factors for metastasis and recurrence were investigated.

**Results:**

A total of 194 patients were included, 154 of whom were female and the average age of all patients was 33 years old. Most patients were asymptomatic, with the most common complaint being abdominal pain or discomfort. Of them, 62 patients underwent parenchyma-preserving pancreatectomy including middle segment pancreatectomy and enucleation, and 132 patients underwent regular pancreatectomy including pancreaticoduodenectomy, distal pancreatectomy and total pancreatectomy. Patients in the parenchyma-preserving surgery group had a shorter duration of operation, less intraoperative bleeding, and decreased risk of combined organ removal and blood transfusion, with no statistical significance yet. The two groups exhibited a similar incidence of postoperative complications including grade B and C pancreatic fistula, delayed gastric emptying, postoperative pancreatic hemorrhage, and other complications, as well as radiological intervention, relaparotomy and the length of postoperative hospital stay. There were no perioperative deaths. All the patients, except 18 of those who discontinued follow-up, were alive with a median follow-up time of 31 months. Three patients in the regular pancreatectomy group were observed to have liver metastasis, and no metastasis was observed in the parenchyma-preserving surgery group. Significant risk factors for tumor metastasis and recurrence were tumor size, angioinvasion, and nerve infiltration.

**Conclusions:**

Parenchyma-preserving surgery did not significantly increase the frequency of perioperative complications or recurrence and might be preferable if comprehensive conditions allow.

## Introduction

Solid pseudopapillary neoplasm (SPN) or solid pseudopapillary tumor (SPT) of pancreas was described by an authoritative pathologist, Virginia Kneeland Frantz, in 1959 [1] and was officially renamed by the World Health Organization (WHO) in 1996. SPN is a relatively rare type of pancreas tumor, accounting for 1–2% of pancreatic neoplasms [2]. It predominantly occurs in adolescent and young adult females and is, thus, called daughter disease [3, 4]. There are no specific symptoms of SPN. With the accumulation of cases and experience from single center studies with small sample size, the knowledge of SPN has improved gradually than before and remains worthy of study. SPN is considered as a low-grade malignant tumor with an indolent growing pattern. Operation is the preferred treatment currently, and its purpose is the removal of the primary lesion in the pancreas and metastatic lesion, without routine lymphadenectomy [5–7]. With a certain potential for recurrence and metastatic dissemination, the recurrence rate is reported at 2.6–3.5% according to a meta-analysis [3, 8]. The prognosis of SPN patients is extremely favorable if followed by complete resection [7]. Patients with recurrence and metastasis may survive for a long time following reoperation. If perioperative safety and efficacy are warranted, surgical methods that preserve pancreatic parenchyma and function might be prioritized. Additionally, although SPN with surrounding tissue invasion, perineural invasion, vascular invasion on microscopic pathology exhibits malignant biological behaviors, no agreement has been achieved on the effect of these characteristics on the recurrence and metastasis. Identification of risk factors would aid in the follow-up of high-risk patients.

In this study, we retrospectively collected and analyzed the clinicopathological data of recent patients with SPN classified into regular pancreatectomy group and parenchyma-preserving surgery group in our center, compared the perioperative safety and outcomes between patients of the two groups, and investigated risk factors for metastasis and recurrence to improve SPN cognition and benefit patients.

## Methods

### Patient population

All patients with SPN undergoing their first operation were screened at pancreas center, the First Affiliated Hospital with Nanjing Medical University from May 2016 to October 2021. All cases were confirmed by pathological examination including hematoxylin and eosin stain and immunohistochemistry. Patients undergoing pancreaticoduodenectomy (PD), distal pancreatectomy (DP), and total pancreatectomy (TP) constituted the regular pancreatectomy group, and those undergoing middle segment pancreatectomy (MSP) and enucleation constituted the parenchyma-preserving surgery group. Other surgical procedures were not performed and collected. The consent form was collected at the time of admission. The study was complied with the Declaration of Helsinki and approved by the Ethics Committee of the First Affiliated Hospital with Nanjing Medical University.

### Surgical procedures

The surgical procedure included PD (classic Whipple procedure or pylorus-preserving pancreaticoduodenectomy, PPPD) with modified one-layer duct-to-mucosa pancreaticojejunostomy [9], DP (with splenectomy, the Warshaw technique, or the Kimura technique), total pancreatectomy, MSP, and enucleation and was established by preoperative diagnosis, individual surgeon preference, and intraoperative discretion depending on tumor location and size.

### Data collection

Demographic data including sex, age, chief complaint, diabetes, history of smoking and drinking, preoperative carbohydrate antigen (CA) 19–9, preoperative carcinoembryonic antigen (CEA), pathological parameters including tumor size (the largest diameter), margin status, Ki-67 index, number of lymph node metastasis, number of detected lymph nodes, angioinvasion, and nerve infiltration, operative parameters including surgical procedure, operation duration, combined organ resection, blood transfusion, and estimated blood loss, and preoperative computed tomography (CT) scans were retrospectively collected from electronic medical records in our hospital. Surgical complications, including postoperative pancreatic fistula (POPF), delayed gastric emptying (DGE), and postoperative hemorrhage (POPH), were assessed and graded based on criteria established by the International Study Group of Pancreatic Surgery [10–12].

### Follow-up

Following discharge, regular follow-up of patients was performed. Follow-up data, including exocrine and endocrine function of pancreas, survival status, and the presence or absence of recurrence and metastasis, were gathered from the hospital records or through regular phone interviews with patients. Endocrine dysfunction included new-onset diabetes or deterioration of preoperative diabetes. Exocrine pancreatic insufficiency was measured in terms of enzyme supplementation or steatorrhea. The last follow-up was in January 2022.

### Statistical analysis

Categorical variables were represented as frequency. Continuous variables were expressed as means ± SD or median (interquartile range). The Student *t* test was used to compare continuous parameters between the two groups. The chi-squared test and Fisher's exact test were used to compare the categorical parameters. Logistic regression analysis was performed to screen the risk factors of metastasis.* P* < 0.05 was considered statistically significant. All statistical analyses were performed using SPSS 26.0 software.

## Results

### Safety and efficacy of parenchyma-preserving procedure

From May 2016 to October 2021, a total of 194 patients undergoing their primary surgery at our center were included in this study. There were 154 females and 40 males, with an average age of 33 years. Abdominal pain or discomfort was the main complaint of patients, and more than half were admitted after a physical examination that revealed a pancreatic tumor (Table 1).

**Table 1:** Demographic and clinicopathological characteristic

Parameter		Total	Regular pancrectomy	Parenchyma preservation	*P* value
		194	132	62	
Sex	Male	40	27	13	0.934
	Female	154	105	49	
Age (mean ± SD)		33.62 ± 13.14	34.49 ± 14.26	31.76 ± 10.19	0.177
Chief complaint	Abdominal pain or discomfort	57	39	18	0.617
	Asymptomatic	134	90	44	
	Others	3	3	0	
Diabetes	Yes	6	4	2	1.000
	No	188	128	60	
Alcohol	Yes	6	6	0	0.179
	No	188	126	62	
Smoking	Yes	8	8	0	0.057
	No	186	124	62	
Preoperative CA 19–9 (IQR) [U/mL]		9.73(6.35–15.27)	9.54(5.88–15.24)	10.71(6.89–15.36)	0.424
Preoperative CEA (IQR) [g/L]		1.13(0.76–1.75)	1.13(0.77–1.71)	1.10(0.69–1.78)	0.898
Surgical procedure	PD/PPPD	36	36	0	
	DP(KIMURA)	95	29	0	
	DP(WARSHAW)		18		
	DP(with splenectomy)		48		
	TP	1	1	0	
	Enucleation	15	0	15	
	MSP	47	0	47	
Tumor Size (mean ± SD)(cm)		5.09 ± 3.01	5.66 ± 3.14	3.73 ± 2.16	0.000 ^*^
Margin	Positive	5	3	2	0.001 ^*^
	< 1 mm	58	50	8	
	Negative	131	79	52	
Ki-67	< 3%	114	77	37	0.743
	3–20%	67	45	22	
	> 20%	2	2	0	
Lymph node metastasis		3	3	0	1.000
Lymph node detection (mean ± SD)		6.17 ± 5.38	7.15 ± 5.50	1.91 ± 1.07	0.000 ^*^
Angioinvasion	Present	3	3	0	0.553
Nerve infiltration	Present	8	7	1	0.283

*PD* pancreaticoduodenectomy, *PPPD* pylorus-preserving pancreaticoduodenectomy, *DP* distal pancreatectomy, *TP* total pancreatectomy, *MSP* middle segment pancreatectomy, *IQR* interquartile range, *SD* standard deviation

^*^p value < 0.05)

The patients were divided into two groups based on the degree of parenchymal preservation in the operation: regular pancreatectomy group including PD or PPPD (19%), DP with the spleen (25%), Kimura technique (15%), or Warshaw technique (9%), and TP (< 1%) and the parenchyma-preserving surgery group including MSP (24%) and enucleation (8%). There was no significant difference in gender, age, main complaint, general condition, and the level of preoperative CA 19–9 and CEA between the two groups (Table 1).

Patients in the regular pancreatectomy group had larger tumor size than that in the parenchyma-preserving surgery group (5.66 ± 3.14 versus 3.73 ± 2.16 cm). More lymph nodes were easily detected in surgical specimens in regular pancreatectomy group (7.15 versus 1.91). However, there was no significant difference in other pathological parameters such as number of lymph node metastasis, Ki-67 index, angioinvasion or nerve infiltration between the two groups (Table 1).

To evaluate the safety of parenchyma-preserving surgery, we compared surgical parameters and the incidence of postoperative complications. Patients in the parenchyma-preserving surgery group had a shorter operation duration (181 min versus 196 min), less intraoperative bleeding (120 mL versus 160 mL), and a lower risk of combined organ removal (0% versus 4.5%) and blood transfusion (1.6% versus 5.3%) than those in the regular pancreatectomy group. However, these values were not statistically significant. In terms of postoperative complications, the incidence of grade B or grade C pancreatic fistula, DGE, and POPF were statistically identical between the two groups, and there was no significant difference in the incidence of other complications, such as biliary fistula, chylous leakage, or incision infection, as well as radiological intervention and the length of postoperative hospital stay. Two patients in each group underwent a second operation. The perioperative mortality rate was zero in both groups. However, there were three readmissions and four readmissions in two groups respectively. Postoperative metastasis was not observed in the parenchyma-preserving surgery group. Furthermore, the incidence of exocrine pancreatic insufficiency and endocrine dysfunction were significantly lower in the parenchyma-preserving surgery group than that in the regular pancreatectomy group (Table 2).

**Table 2:** Intraoperative data and complication

`		Total	Regular pancreactomy	Parenchyma preservation	*P* value
		194	*n* = 132	*n* = 62	
Duration of operation (mean ± SD)		191 ± 78	196 ± 86	181 ± 59	0.244
LN dissection	Yes	76	52	24	0.927
	No	118	80	38	
Combined organ resection	Yes	13	12	1	0.065
	No	181	120	61	
Blood transfusion	Yes	8	7	1	0.283
	No	186	125	61	
Blood loss (mean ± SD)		147 ± 183	160 ± 201	120 ± 132	0.157
Complication					
POPF					0.709
	Grade A/absent	75	53	22	
	Grade B + C	119	79	40	
DGE					1.000
	Absent	190	129	61	
	Present	4	3	1	
POPH					0.386
	Grade A/absent	188	129	59	
	Grade B + C	6	3	3	
Other		20	12	8	0.415
Radiological intervention		9	4	5	0.213
Relaparotomy		4	2	2	0.594
Postoperative hospital stay(IQR)		10(9–13)	10(9–13)	11(9–12)	0.768
Mortality		0	0	0	
Readmission		7	3	4	0.148
Metastasis		3	3	0	0.553
Exocrine pancreatic insufficiency		29/176	25/116	4/60	0.011 ^*^
Endocrine dysfunction		31/176	29/116	2/60	0.000 ^*^

*IQR* interquartile range, *SD* standard deviation

^*^p value < 0.05)

### Demographics and clinicopathologic features subgrouped by age

Considering the difference in the incidence of SPN in patients of different ages, we compared the demographics and clinicopathological features of the patients divided into three groups based on age. As indicated in Table 3, there was no difference in the distribution of symptoms, sex, and preoperative CA 19–9 and CEA, whereas there was a difference in the distribution of diabetes, which was consistent with age. The composition ratios of surgical procedure in each group are slightly different. There was no significant difference in the lymph node metastasis, angioinvasion, nerve infiltration, and postoperative metastasis of the groups. However, Ki-67 revealed a statistically significant difference.

**Table 3:** Subgroup analysis by age

		Subgrouped by age			
		< 18	18– < 40	> 40	*P* value
		22 cases	114 cases	58 cases	
Age (mean ± SD)		13.36 ± 2.17	29.25 ± 5.84	49.88 ± 7.01	
Sex	Male	5	20	15	0.464
	Female	17	94	43	
Chief Complaint	Abdominal pain or discomfort	12	83	39	0.149
	Asymptomatic	9	31	17	
	Others	1	0	2	
Diabetes	Yes	0	1	5	0.031 ^*^
Preoperative CA 19–9* (IQR) [U/mL]		8.06(5.01–11.65)	9.89(6.27–14.82)	11.32(6.70–16.40)	0.139
Preoperative CEA* (IQR) [g/L]		0.81(0.64–1.25)	1.04(0.70–1.64)	1.45(1.04–2.28)	0.114
Surgical procedure	PD/PPPD	4(18%)	19(17%)	13(22%)	0.046 ^*^
	DP	11(50%)	51(45%)	33(57%)	
	MSP	3(14%)	35(31%)	9(16%)	
	TP	1(5%)	0(0%)	0(0%)	
	Enucleation	3(14%)	9(8%)	3(5%)	
Tumor size (mean ± SD) (cm)		6.28 ± 3.11	5.12 ± 3.00	4.58 ± 2.90	0.096
Margin	Positive	0	3	2	0.810
	< 1 mm	5	36	17	
	Negative	17	75	39	
Ki-67	< 3%	9	63	42	0.026 ^*^
	3–20%	12	41	14	
	> 20%	1	0	1	
Lymph node metastasis		0	2	1	1.000
Angioinvasion	Present	0	2	1	1.000
Nerve infiltration	Present	1	5	2	1.000

(*PD * Pancreaticoduodenectomy, *PPPD * Pylorus-preserving pancreaticoduodenectomy, *DP * Distal pancreatectomy, *TP * Total pancreatectomy, *MSP * Middle segment pancreatectomy, *IQR * Interquartile range, *SD * Standard deviation. ^*^ represents *p*-value < 0.05)

### General and misdiagnosed image features

**Fig. 1:**
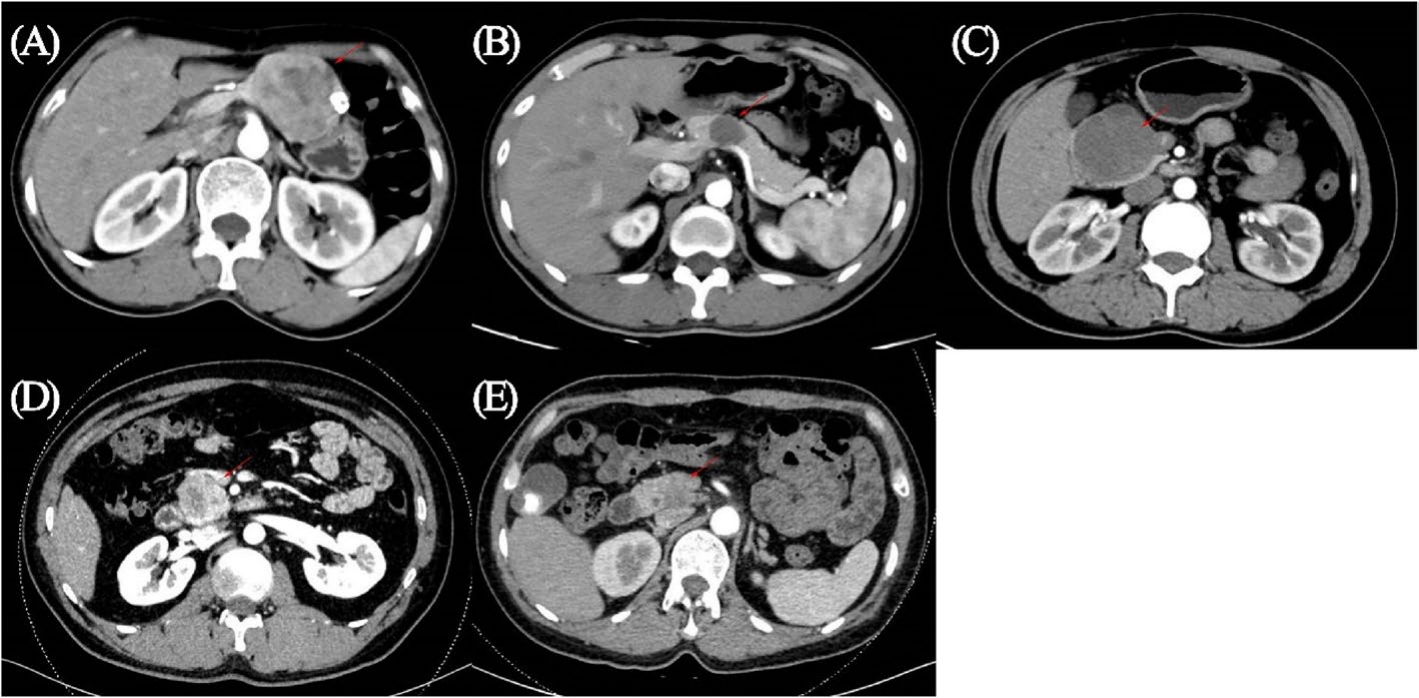
SPN with different proportion of cystic and solid components. **A** Characteristic solid and cystic lesion, **B** pure cystic lesion, **C** mostly cystic lesion, **D** mostly solid lesion, **E** pure solid lesion

**Fig. 2:**
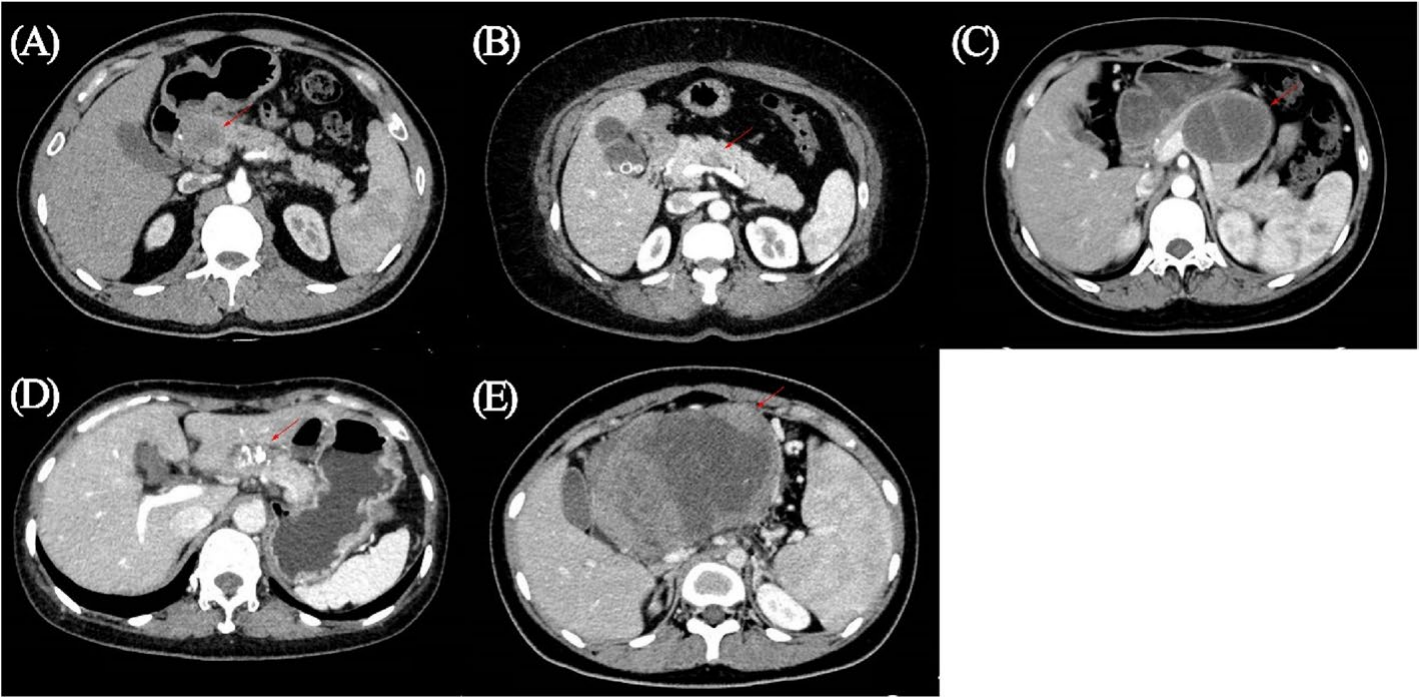
Atypical and easily misdiagnosed SPN images. **A** A 33-year-old-female, low density lesion in pancreatic head with fuzzy boundary, like PDAC. **B** A 45-year-old-female, small solid lesion in body of pancreas, like neuroendocrine tumor. **C** A 19-year-old-female, pure cystic lesion with septa inside, like mucinous cystic neoplasms. **D** A 49-year-old-female, several calcifications around the lesion, like chronic pancreatitis. **E** A 14-year-old female, huge mass with portal vein contour irregularity

One hundred eleven abdominal CT scans were available and reviewed at our hospital (Table 4). Execpt for a case that could not be evaluated by CT scan, there were 40 cases of mass in the head of the pancreas, 13 in the neck of the pancreas, and 57 in the body and tail of the pancreas. Based on the proportion of cystic and solid components (Fig. 1), SPN on the scan displayed pure cystic (*n* = 3), mostly cystic (*n* = 9), cystic and solid or mixed density (*n* = 81), mostly solid (*n* = 11), or pure solid components (*n* = 6). Calcification was present in different site of tumor. About two-thirds of cases were radiographically typical and had high diagnostic values. The rest of the images were not typical, and the diagnosis of SPN was not a priority, which requires the expertise of experienced specialists. The scan might be misdiagnosed as pancreatic ductal adenocarcinoma (PDAC), pancreatic neuroendocrine tumor (PNET), or mucinous cystic neoplasm (MCN) when SPN displayed pure solid or pure cystic components (Fig. 2A–D). In addition, SPN could showed extremely large size and contacted with several organs, which is indistinguishable in the organ origin of the tumor radiographically (Fig. 2E).

**Table 4:** Image presentation of SPN

Preoperative CT image		
Location	Head	40
	Neck	13
	Body and tail	57
Component proportion	Pure cystic	3
	Mostly cystic	9
	Cystic and solid	81
	Mostly solid	11
	Pure solid	6
Calcification		51

*CT* computed tomography

### Risk factors for recurrence and metastasis

Follow-up data for 176 patients were available, and 18 patients were lost to follow-up. All the surviving patients had a median follow-up duration of 31 months (3 − 69 months). Three female patients presented with liver metastasis at 6, 12, and 39 months, respectively, following the initial operation. These patients underwent radiofrequency ablation, and one of them had a simultaneous liver resection with relapse. Clinicopathological risk factors for metastasis were investigated. Significant risk factors for tumor recurrence and metastasis were tumor size, angioinvasion, and nerve infiltration (Table 5).

**Table 5:** Risk factors for metastasis of SPN

	Univariate analysis
Factor	*P* value
Sex	0.998
Age	0.934
Preoperative CA 19–9	0.352
Tumor size	0.003 ^*^
Margin	0.955
Ki-67	0.265
Lymph node metastasis	0.999
Angioinvasion	0.006 ^*^
Nerve infiltration	0.045 ^*^

^*^p value < 0.05)

## Discussion

Solid pseudopapillary neoplasm of the pancreas is a rare pancreatic tumor accounting for 1 − 2% of all pancreatic tumors. Since its discovery in 1959, public knowledge of this disease has steadily increased.

The average age of onset of SPN is 27 years in the Chinese population, mostly affecting young people [3]. Patients older than 45 years have been reported to constitute only a small percentage of all SPN cases [6]. In our study, patients with ages < 40 years accounted for 70% of all cases, consistent with data in Germany. Kang et al. correlated tumor size with the age of patients and observed that older patients had relatively smaller tumors than younger patients [13], which was not directly found in our patients. However, older patients had lower Ki-67 index, and there was no significant difference in the margin lymph node metastasis, angioinvasion, nerve infiltration, and postoperative metastasis among patients subgrouped by age. SPN predominately occurs in female whereas males are rarely affected [7, 14]. Our study appears to have a little larger number of male patients than previously reported [3, 14]. Female preponderance could be explained by proximity to the ovarian ridge during development and the presence of progesterone receptors in malignant cells [5, 15]. Patients with SPN usually lack specific manifestations. Non-specific abdominal pain and discomfort and incidentally detection are common chief presentation of patients. Other chief complaint included nausea and vomiting, obstructive jaundice, and discovery in a laparotomy due to abdominal trauma in our cases.

Preoperative image has become the most commonly used tool for diagnosing SPN. The advantages of multidetector CT and magnetic resonance imaging are non-invasion and convenience [16]. SPN on CT is typically characterized by a well-defined, low-attenuation mass with peripheral enhancement and complex cystic components. Besides, necrosis and internal hemorrhage may coexist [17]. However, not all lesions include both solid and cystic components [18–20]. Based on our findings, SPN on the image may also present pure cystic component, largely cystic and rarely solid component, largely solid and rarely cystic component, or pure solid component. These atypical lesions may characterize as PNET, MCN, PDAC, gastrointestinal stroma tumors, or other unspecified solid tumors [21, 22]. Calcification is also reported in the tumor, and its presence may aid in diagnosis [3]. Recently, radiomics has also been applied in the differential diagnosis of SPN [23, 24]. In addition, endoscopic ultrasound is useful for differentiating SPN from other tumors [25]. Combination with fine needle aspiration could increase the pre-operative diagnostic yield of SPN [26], without increasing the risk of metastasis and recurrence [27].

For the treatment for the patients with SPN, operation is a priority for the primary and metastatic lesion. Routine lymphadenectomy has been shown to be unnecessary for the disease [5–7]. In recent years, the incidence of parenchyma-preserving surgery has increased, which means endocrine and exocrine function are more reserved [13]. Liu et al. compared enucleation with conventional pancreatectomy [19]. They observed that enucleation had a shorter operation duration, less blood loss, lower rate of exocrine insufficiency, and comparable morbidity, with no increased risk of tumor recurrence than conventional pancreatectomy. In this study, we included patients experiencing enucleation and MSP in the parenchyma-preserving surgery group. This classification was appropriate since pancreatic parenchyma was utmost preserved with enucleation and MSP. We found patients in parenchyma-preserving surgery group experience less endocrine insufficiency containing diabetes deterioration and new-onset diabetes mellitus and has less complaints of fatty stools and unintentional weight loss reflecting exocrine insufficiency. Even in this case, control of risk factors for diabetes mellitus as well as early detection and management may reduce the burden of endocrine insufficiency and its complications [28]. Meanwhile, pancreatic enzyme supplementation, especially correct dosing or intake of pancreatic enzymes, contributes to the prevention of exocrine insufficiency [29]. Besides, compared with those in the regular pancreatectomy group, patients in the parenchyma-preserving surgery group had smaller tumor sizes and fewer lymph nodes, with no significant difference observed among the other pathological parameters including the number of lymph node metastasis, Ki-67 index, angioinvasion, or nerve infiltration. Moreover, the incidence of perioperative complications and recurrence did not increase in the parenchyma-preserving surgery group. Furthermore, the adoption of minimally invasive techniques increased [13]. Data of Mou et al. revealed that laparoscopy DP for SPN had short-term benefits including first flatus time, diet onset time, and postoperative hospital stay compared with open DP [30]. Long-term outcomes of laparoscopic DP were identical to those of open DP. This is consistent with another single center [31]. Robot approach is likely to be useful in laparoscopic parenchyma-preserving surgery and more experience of robot-assisted pancreatectomy are needed. Based on our experience and existed studies, surgical choice is determined by a combination of several factors. Preoperative diagnosis should be as precise as possible. In the condition that preoperative imaging is highly suspected of SPN or pathology results have been obtained, parenchyma-preserving pancreatectomy might be possible. If a patient is diagnosed preoperatively with SPN or pancreatic cancer without pathological evidence, regular pancreatectomy and routine lymph node dissection, rather than parenchyma-preserving surgery, might be performed for safety. In addition, it is noteworthy that the final surgical strategy may be influenced by abdominal exploration and the surgeon's preference and experience [6]. The location and size of tumor have influence on surgical choice. For example, if tumor located in pancreatic head is too large or close to the intrapancreatic bile duct, parenchyma-preserving surgery might not be performed due to increased risk of biliary and pancreatic fistula. For tumor located in the neck or body, parenchyma-preserving surgery is more likely to be performed. Middle segment pancreatectomy is a prior choice if residual parenchyma of pancreas distal to the expected left pancreatic transection plane was enough. Enucleation could be performed when tumor located on the surface of the pancreas and is not closely related to the pancreatic duct. Thus, parenchyma-preserving surgery and a minimally invasive approach may be preferable if comprehensive conditions allow. Adjuvant chemotherapy or radiotherapy is an alternative for patients when the operation is unsuccessful [7]. However, no consensus has been achieved, and additional evidence is required to justify the role of adjuvant radiotherapy or chemotherapy.

The prognosis of patients with SPN is quite favorable with 5-year and 10-year survival rates [31]. The surviving patients had a median follow-up duration of 31 months in this study. However, the probability of recurrence and metastasis remained to exist. The incidence of recurrence and distant metastasis varies among reported cohorts, ranging from 0% to more than 10% [20, 21]. Two large sample-sized meta-analyses revealed recurrence rates of 2.6% and 3.5%, respectively. Within the first 5 years following operation, SPN most frequently recurs and spreads to distant organs, with the liver being the most common site [8]. In our study, except for 18 patients who lost follow-up, three patients (1.7%) had a relapse at 6, 12, and 39 months, respectively, following initial operation. All three patients had metastatic lesions in the liver and underwent radiofrequency ablation. One of them relapsed and required liver resection. No deaths owing to relapse were recorded among them at the last follow-up. No consensus has been reported on risk factors for recurrence and metastasis so far. The most addressed pathological risk factors were tumor size and the Ki-67 index. Overall, larger tumor diameter predicted a poor prognosis with different cutoff value [13, 32]. A meta-analysis by Fu et al. indicated that the gradual increase in Ki-67 index is associated with the risk of malignancy in SPN [33] and Ki-67 > 4% might be an adverse prognostic factor in their own data [34], which is inconsistent with classification for pancreatic neuroendocrine neoplasia. Our study observed tumor size as a risk factor for metastasis rather than the Ki-67 index. Besides, recurrence might be more common when tumors are associated with other pathological features including lymph node metastasis, tumor cell infiltration of the peripancreatic adipose tissue, lymphangion invasion, neural invasion, and tumor necrosis [13, 21, 35]. Angioinvasion and nerve infiltration were risk factors of metastasis based on our data. However, margins status is not a risk factor [36], consistent with our observation. In patients with underlying risk factors for recurrence and metastasis, a relatively long follow-up is recommended.

To our knowledge, this is one of the few single-center retrospective studies on SPN comparing perioperative safety and outcome of patients classified based on parenchyma-preserving pancreatectomy and regular pancreatectomy. We also evaluated CT images of SPN to improve diagnosis and analyzed risk factors predicting metastasis and recurrence of SPN to guide follow-up. However, this study had several limitations. All data were collected retrospectively from a single center. The inherent bias of retrospective collection was inevitable and the results might be influenced by center-specific practices. Due to small number of metastasis, several risk factors for metastasis might not expose in this study. Moreover, the two groups in this study were based on whether the operative method itself preserved the parenchyma or not. The extent of parenchyma preservation was not quantified. Patients were not equally clarified into two groups. Fox example, patients with large tumor located in pancreatic head or patients with tumor close to the splenic hilum could only undergo regular pancreatectomy. This might reduce comparability to some extent. Other limitations of our study included a paucity of data on long-term complications and molecular pathogenesis. Overall, these limitations indicate that more comprehensive facts should be studied further.

Conclusively, parenchyma-preserving surgery did not significantly increase the incidence of perioperative complications and recurrence and is preferable if comprehensive conditions permit.

## Data Availability

Data supporting the findings of this study are available from the respective authors upon reasonable request.
